# International workshop on “professionalism in the practice of medicine- where are we now?”

**DOI:** 10.1186/s13584-017-0144-5

**Published:** 2017-04-03

**Authors:** Ora Paltiel, Lior Lowenstein, Jonathan Demma, Orly Manor

**Affiliations:** 1 Braun School of Public Health and Community Medicine, Hadassah-Hebrew University, Jerusalem, Israel; 2 Department of Obstetrics and Gynecology, Rambam Health Care Campus, Haifa, Israel; 3 Ruth and Bruce Rappaport Faculty of Medicine, Technion, Haifa, Israel; 4 Department of Surgery, Hadassah-Hebrew University Medical Center, Jerusalem, Israel; 5 National Institute of Health Policy Research, Gertner Institute, Tel Hashomer, Israel

**Keywords:** Professionalism, Medical education, Peer support, Complaints, Digital age, Israel

## Abstract

Medical professionalism refers to attributes, values, behaviors, responsibilities and commitments of physicians that are congruent with the public’s expectations. An international workshop on medical professionalism took place at the Dead Sea, Israel, on December 11–12, 2016. The meeting brought together local medical professionals, physicians and others, as well as international experts, to discuss definitions of professionalism and wrestle with current challenges facing the profession including its perceived status and physician satisfaction, unprofessional behavior and its relation to health care quality and patient safety, and professionalism as a learned competence. Individual medical schools reported on educational efforts to promote professionalism in their curricula. Patient complaints as an improvement mechanism were explored on a national and health plan level. I was found that complaints regarding physician behavior are rare in the Israeli context and need to be dealt with expeditiously at a local level in order to be effective tools for change. The meeting provided a venue to understand local and international strategies and mechanism for regulation and self-regulation, highlighting the role of the Israel Medical Association. A major focus of the meeting was on intergenerational differences in attitude and practice and the necessary adaptation of medicine to the digital age. We provide an overview of the topics addressed, synopsis, evaluation and lessons learned in this first-ever national meeting on medical professionalism in Israel.

**Electronic supplementary material:**

The online version of this article (doi:10.1186/s13584-017-0144-5) contains supplementary material, which is available to authorized users.

## Introduction

Professionalism, according to the Merriam-Webster’s Learner’s Dictionary, is defined as “the skill, good judgment, and polite behavior that is expected from a person who is trained to do a job well” [1]. In 2002, the American Board of Internal Medicine (ABIM) published a physician charter on medical professionalism [2], including three fundamental principles- the primacy of patient welfare, patient autonomy and social justice- and 10 professional responsibilities and commitments ranging from professional competence, honesty and maintaining patient confidentiality to societal obligations including improving quality and access to care. The charter has since been ratified by scores of medical organizations the world over [3], but not by the Israel Medical Association (IMA). The reasons for the latter are unclear (see below). Small scale events had previously been held in Israel on the topic of Professionalism (eg. visit by Drs.Y. Steinert and Cruess [4], Beersheva, 2006). The current workshop, which took place on December 11–12, 2016 at the Dead Sea is the first on a national scale in Israel. The event, described below, provided an exceptional opportunity to tackle crucial and under-discussed aspects of medical practice and training in an intimate setting. The meeting was organized and funded by the Israel National Institute for Health Policy Research (NIHPR), which periodically organizes international workshops on current topics such as Big Data, Hospital Funding [http://www.israelhpr.org.il/e/] etc. and is instrumental in bringing both national and international players together for serious discourse on burning important issues of health care. It is also the sponsor of the IJHPR, in which this meeting report is being published.

The workshop was exceptional in many ways, including the array of professions and ranks represented. Fifty physicians, nurses, social workers, psychologists and biostatisticians came together for two full days to engage and grapple with issues of physician behavior, patient expectations and changing paradigms of professionalism. Participating physicians represented both primary and community care as well as hospital sectors (12 institutions country-wide) including specialists in family medicine, internal medicine, general surgery, ENT, obstetrics and gynecology, neurology, histo-pathology, geriatrics, psychiatry and medical administration. All four Israeli health plans were represented as well as the country’s five medical schools. Participants ranged in age from early 30s to the 80s, with a median of 27 years in practice (Table 1).

**Table 1:** Specialties and affiliations of meeting participants

Professional areas and specialties represented	Institutional affiliations of participants
Family Medicine Internal Medicine Geriatrics, Hematology, Endocrinology Pediatrics Ob-Gyn General Surgery Otolaryngology Neurology Psychiatry Medical Administration Nursing Social Work Psychology Biostatistics	Israeli Universities: Bar Ilan University, Technion, Tel Aviv University, Hebrew University-Hadassah, Ben-Gurion University Non-Israeli Universities: Harvard, UCL, University of Toronto, Durham University Israeli National Health Plans: Clalit, Maccabi, Meuhedet, Leumit Health Services Hospitals in Israel: Poria, Carmel, Rambam, TA-Souraski, Barzilai, Hadassah Ein Karem, Hadassah Mt. Scopus, Shaare Tzedek, Soroka Non-Israeli hospitals: Brigham and Womens’, Boston; Mount Sinai, Toronto. Ministries, organizations: MOH, BMA, National Insurance institute, NIHPR

The organizers hoped the workshop would not only shed light on problems but also provide a forum to explore solutions. The following report highlights the content, main issues and controversies raised, feedback, and lessons learned from the meeting.

The topics addressed at the meeting included:

- Definitions of professionalism
- Models and systems that have taken on the challenge of professionalism
- Professionalism as a learned competence
- Whether complaints can serve as tools to improve professionalism
- Models of self-regulating professional behavior
- Generational issues and professionalism in the digital age.

The meeting agenda (Additional file 1) addressed these topics sequentially.

## Synopsis

The program was opened by Orly Manor, Chair of the Board of the NIHPR and Moshe Bar Siman Tov, Director-General of the Ministry of Health (MoH). Both emphasized the importance of professionalism to medical practice and to the Israeli public. Prof. Manor recounted personal experiences with unprofessional behavior and raised the issue that Israel’s public is concerned about receiving the best standard of care in times of severe illness.

In the introductory lecture, Ora Paltiel pointed out that while there are now over 20 definitions of professionalism, most listing a group of principles, attributes, and behaviors, common to these definitions are aspects concerning competence, trust, appropriate relationship between physicians and patient and avoidance of conflict of interest. For its part the ABIM charter [2] includes a social commitment to just distribution of finite resources, improving access to care and improving quality of care. OP noted that in the Israeli context, where universal health care is considered a right, these “social-contract” aspects of professionalism, such as universal access to care, improving access to care, just distribution of finite resources, are generally viewed by physicians and the public as the role of the health system as a whole, as stipulated in the National Health Law, rather than the personal responsibility of individual physicians. The American Board of Medical Specialties (ABMS) defines medical professionalism as a “belief system in which group members (‘professionals’) declare (‘profess’) to each other and the public the shared competency standards and ethical values they promise to uphold in their work and what the public and individual patients can and should expect from medical professionals” [5]. The terms “beliefs system” and “profess” add moral overtones to this definition. An important element of the ABMS definition is the focus on meeting the expectations of the individual patient and the public at large.

How do we know whether these expectations are being met? In Israel, surveys by the Central Bureau of Statistics and the Myers-JDC-Brookdale Institute reveal high public satisfaction with the primary care system, including the attitude and the availability of physicians [6, 7]. Surveys by the MoH reveal similar satisfaction levels among those discharged from emergency rooms regarding affability and respectful behavior of physicians and the staff in general [8]. However 66% reported that pain had not been adequately addressed and 55% that physicians did not introduce themselves (although this is required by law). These findings point to the need to address professionalism issues in the Israeli setting.

The session brought an international perspective on current issues of professionalism. Prof. Pali Hungin, President of the British Medical Association, former Dean of Medicine at Durham University, gave a thought-provoking session regarding the current crisis faced by the profession, due in part to changing demands and expectations of the public, on the one hand, and the relative conservatism of the profession on the other. He delineated the symptoms of this crisis, including attrition/dropout, burnout and even decreased enrollment in UK medical schools, and suggested some of their causes - including loss of status, time pressure and loss of clinical autonomy. Prof Hungin warned that Medicine as an esteemed profession is threatened because of changes both in societal expectations and rapid technological transformation. Despite genuine cause for concern, he predicted that as we learn to use digital technologies to their full potential, and as we redefine expertise, medicine will evolve. The paradigm will shift and Prof Hungin foresees enhanced professionalism, increased professional satisfaction, and improved patient outcomes. Change will come because it is necessary.

Prof. Jo Shapiro, an otolaryngologist at Harvard Medical School and Director/Founder of the Center for Professionalism and Peer Support at the Brigham and Women’s Hospital [9, 10] spoke about the necessity of a culture of trust, emphasizing the importance of teamwork, communication, response to errors in a non-threatening way (putting an end to the “shame and blame” culture) and physician wellness. Prof. Shapiro stressed the primacy of mutual respect and trust among clinicians in order to enhance professionalism and combat physician burnout and suicide.

Prof. Shimon Glick from Ben Gurion University (BGU) surveyed the changing views of professionalism in time and place in the medical literature. From a rarely-addressed subject of discourse which mainly focused on its sociological aspects twenty years ago, “professionalism” has now become a common and popular subject for study and debate, with much medical writing these days devoted both to its ethical and educational components. This suggests that in the last twenty years the profession has become more reflective as to the “hows” of practice, in addition to focusing on knowledge-based and technological advances.

An entire session was devoted to professionalism as a learned competence. In the past communication skills were thought to be innate, yet sufficient data and experience have been accumulated to show that they are teachable. Similarly, elements of professionalism, especially behavioral issues such as honesty, confidentiality and enhancing patient autonomy can be learned. Shiphra Ginsburg, Professor of Medicine at the University of Toronto (and co-editor of the current JAMA series on Professionalism [11]) presented data supporting the “teachability” of these behaviors, and introduced the concept of the “hidden curriculum”. Challenging the audience to consider dilemmas in professionalism and medical education, she raised the question whether residents who exhibit behaviors which may be personally detrimental (such as non-adherence to duty hours) may be unconsciously encouraged to do so by their teachers. Dr. Ruti Margalit from The MSR Simulation Centre at Tel Hashomer Hospital reviewed the usefulness of simulation in enhancing professional skills such as communication, end of life discussions, and conveying bad news. The experience of erring in a friendly and non-threatening environment, giving and receiving feedback and debriefing have an important role in enhancing professional skills. The session included a report from each of the Israeli medical schools regarding issues of professionalism, which clearly needs to be a major focus of medical curricula [12]. For instance,

- The Faculty of Medicine of the Galilee (Bar Ilan University) exposes preclinical students to populations with special needs, then in the clinical years students are tasked with explaining the discharge letter and doing a follow up home visit on hospitalized patients. Dr. Peter Gilbey, described longitudinal courses taught in medical humanities and clinical skills which emphasize ethical and professional behavior as well as dyadic and group mentoring programs which encourage discussions on professionalism.
- Prof. Dorith Shaham, Hadassah-Hebrew University (H-HU), described their “Man and Society” course and its professionalism-related content, including cultural competence, medical ethics, sexuality and doctor-patient communication. She described a consensual honor code composed by the students which defined behaviors such as altruism, commitment to excellence, integrity, cultural competence, recognition of the limits of one’s competence, self-awareness, social accountability as defining students’ professional behavior. Despite this, she described the phenomenon of students cheating on examinations as “widespread” and recounted that raising the issue of students working throughout their medical studies as a possible impediment to professionalism was met with great anger and resentment.
- Prof. Nathaniel Laor described multiple curriculum initiatives from the Department of Medical Education at Sackler Faculty of Medicine at Tel Aviv University (TAU) with the goal of helping students to acquire and maintain professional competencies. This includes longitudinal courses on medical education and communication, and providing opportunities for videotaped simulated patient encounters in order to improve communication skills. Current efforts focus on evaluating student performance and professionalism in Internal Medicine departments as well as improving professionalism, and preventing burnout in the context of one of the TAU surgical departments.
- The curriculum of the Technion Medical School in Haifa was presented by Prof. Lior Lowenstein. He described the honor agreement signed by the students which includes respect for patients, peers, team members, and a commitment to honesty and altruism. He also outlined other initiatives including the “Becoming a Physician” course, exposure of students to diversity, multiculturalism and patients from disadvantaged background, as well as training courses on professionalism for mid-career physicians.
- Prof. Glick outlined ongoing teaching throughout the curriculum of BGU dealing with professionalism, including introductory ethics teaching in the first weeks, an ethical code composed by students, early patient exposure, a course in humanities, experiential contact with patients with disabilities, as well as injection of teaching “moments” on professionalism during clinical clerkships, emotional processing, among others.

The session ended with a panel discussion focusing on educational aspects of professionalism training and student responses to these initiatives, such as the writing of student charters for professionalism in each medical school. While many educators feel that the dual commitment to work and study, so common among Israeli students, harms exposure hours and may delay the acquisition of a professional identity, as noted, one of the panel members emphasized that the mere raising of this issue raised the ire of students.

Complaints can be a window into unprofessional behavior, or into gaps between physician behavior and patients’ expectations, and can be predictive of malpractice risk [13]. The workshop explored whether patient complaints are surrogate measures of a lack of professionalism and whether they can be used as tools to enhance it . Two past and one current ombudsmen of the MoH gave an overview of their activities and interventions. One of the previous officials, Prof. Shimon Glick argued that complaints at the level of the MoH are not useful tools to enhance professionalism as they are too far removed from the actual point of care and should have been dealt with at the local level. On the other hand Prof. Chaim Hershko, in a filmed presentation, gave three examples where professional issues (including perceived “unnecessary” and repeated visits to the emergency room for the same complaint, and lack of empathy, privacy and continuity of care for terminally ill patients) were actually dealt with using policies, directives or conferences devoted to these issues at the MoH level.

In the second part of this session the chief physicians/representatives of Israel’s four national health plans which cover the entire civilian population (Yair Birnbaum- Clalit; Avi Porath- Maccabi; Yoav Yehezkeli- Meuhedet; Gershon Segal- Leumit) reported on their organization’s experience regarding patient complaints about professionalism issues or physician behavior. Conference participants were surprised to learn that across the health plans only 5–7% of complaints from the public referred to these issues; while complaints regarding services, entitlements etc. are much more common. Exploring the background of these complaints, the health plans found that although some definitely indicated unprofessional physician behavior, many had to more to do with system issues such as overloading a doctor’s schedule or poor cultural fits between the doctor and the community they were serving. In many cases, administrative intervention eased these tensions and alleviated these structural problems. The paucity of complaints regarding professionalism may point to a lack of a culture of complaint, low expectations or public perceptions that there is “no point” complaining; alternatively this may reflect general consumer satisfaction as reflected in multiple national surveys. While dealing with patient complaints is important, the speakers stressed that proactive measures by the Israeli health plans to enhance quality and professionalism are more productive. There was general agreement that complaints about physician behavior are best handled locally and as expeditiously as possible.

Within the context of technological change and its effects on clinical care the participants heard Dr. Nissan Perez, former curator of photography for the Israel Museum, who offered a fascinating lecture on the early origins of medical photography. Dr. Perez described the rapid uptake of photography as a new technology in the 1800s for uses in consultation, medical teaching or by curiosity seekers. The lecture punctuated the relationship between medicine and society, technology and the humanities.

Self-regulation has traditionally been viewed as a defining characteristic of “free” professions, leading George Bernard Shaw, to quip that, “all professions…. [are]….a conspiracy against the laity” [14]. The second day began with a discussion on self-regulation of physician behavior. Prof. David Katz from University College London described the evolution of the concept of “fitness to practice” and the various self-regulating bodies including the General Medical Council and the Medical Practitioners Tribunal Service (MPTS). He reviewed some of the infamous cases in the UK, including the Bristol, Alder Hey and Shipman incidents [15], which have heightened the involvement and interest of the public in these issues. There has been a rapid increase in complaints from the public since 2008, 30% of which were for competency issues. He described the current separation of investigative, regulatory and adjudication roles in the MPTS. Prof. Katz reminded us that the self-regulation of the medical profession is conferred on the basis of public trust that physicians will do the job by always putting the patient’s interests first. Dr. Tami Karni, head of the Ethics Bureau of the IMA provided an overview of the remit of this office in cases of professionalism and ethics lapses, citing some concrete examples, including inappropriate sexual relations between physicians and patients, problematic use of the internet by physicians for political posts, and physician advertising. She outlined the role of the IMA in publishing guidelines and ethical codes as well as dealing with individual issues, their investigation, publication and sanctions involved. Prof. Jo Shapiro, reiterating the relationship between professionalism, patient safety and healthcare quality, described a proactive mechanism in her hospital carried out by the Center she directs in dealing with unprofessional, disruptive behavior [9, 10]. She showed a video of a simulated case demonstrating recognizable bullying behavior on the part of an obviously otherwise competent and well-regarded surgeon. The video engendered active discussion, and Prof. Shapiro encouraged the audience to look for the “outliers” in their own institutions, and to find ways to remediate, with the support of the administration. She showed that although remediation is often possible (58%), some of the identified outliers do eventually leave the institution (11%).

Many questions were addressed to her regarding institutional funding, backing and support for these types of initiatives, and she described the processes required to actualize them. The main argument used to garner the support of the administration is that these activities enhance patient safety and quality of care, and can be cost- and reputation-saving in the long run.

The last formal session of the workshop was entitled “Professionalism in the Digital Age”. Professor Shmuel Reis, director of Medical Education at H-HU outlined both the challenges and the potential advantages of digital technologies and communication in medical care. One such challenge is the blurring of boundaries between personal and professional lives. He stressed that the unavoidable encounter between digital media and medicine will raise new questions regarding professionalism, physicians’ reputations and required competencies. Dr Orit Karnieli-Miller of TAU presented original research demonstrating the effect of computers and physician behavior in medical encounters [16], including the enhanced placebo effect engendered by eye contact and improved communication. The lecture showed that high level research, both qualitative and quantitative, is currently carried out in Israel investigating issues related to professionalism.

In the final panel on generational issues in professionalism, Prof. Ziv Gil (Technion), presented results of a 2008 survey of attitudes of physician from the silent, baby-boomer, X and “millennial” generations, these defined by their birth year and relationship to digital technologies [17]. He pointed out differences in learning styles, compliance with rules, and attitudes toward the profession across the generations. He also presented reforms at his own department aimed to enhance patient-centeredness, remove barriers to communication etc. in response to changing patient expectations of the medical profession.

Members of the panel, Drs. Orna Reichman, Rotem Telem and Sameer Kassem spoke about the private-public mix, changing attitudes among the generations and the importance of transparency and involvement of trainees in scheduling and setting educational goals for young residents. Dr. Reichman was questioned over the influence of the availability of private medicine within public institutions (“Sharap”) and her response was that this arrangement did not affect professionalism, although this opinion was not shared by all. On the other hand, one participant queried whether the IMA’s failure to officially ratify the ABIM charter was due to its reluctance to criticize or minimize the practice of private medicine in Israel. While general concern has been voiced worldwide [18, 19] about the effect of restricted duty hours on professionalism, Dr. Kassem stressed that duty-hour regulations are not an excuse for leaving clinical tasks undone, and that most residents manage to comply with these regulations while completing or successfully handing over their professional duties.

He final event in the Workshop was a lively interactive session involving 12 clinical vignettes dealing with professionalism lapses and issues such as honesty, confidentiality, support for depressed physicians, inappropriate use of the internet etc. These issues were discussed in groups of up to 10 participants. Group rapporteurs summarized the discussions in the plenary. Summing up this session, Prof. Ginsburg emphasized both the universality of the vignettes (the impaired or depressed physician, confidentiality, etc.) and the unique aspects of Israeli medicine (eg. the political context, medical practice during armed conflict) which these scenarios raised.

## Evaluation and lessons learned

Formal feedback was sought from all participants who were asked to answer a structured questionnaire as well as provide free-text comments. This kind of evaluation is uncommon in Israeli medical conferences, but it provided important data as to the impact of the meeting. Apart from the positive evaluation of the technical and organizational aspects of the meeting, participants (62% response rate) noted they had attended a very important workshop which would have an immediate influence on their own practice and teaching. Participants felt it was noteworthy that very senior physicians in the Israeli health care system (MoH and Health Plans) along with their more junior colleagues attended and participated throughout, stressing the relevance and importance of the subject in the local context. The suggestion that more simulation be used to teach professionalism skills was met with enthusiasm mixed with concern as to who would fund this.

This meeting brought together a country-wide representation of physicians from all levels of the medical hierarchy including the highest echelons from the Health Plans and MOH as well as more junior physicians, trainers, teachers and other professionals to grapple in a meaningful way with issues that cut across medical specialties, and thus should lead to dissemination and more generalized discussion of the issues raised. The workshop brought to light projects currently undertaken by Israeli organizations (universities, individual hospitals and health plans) with regards to medical professionalism. In order to implement changes in a standardized way participants called on the MOH to include professionalism in its next strategic working plan as well as urging the IMA to ratify the ABIM charter.

Dissemination and education are key to raising awareness of the challenges regarding professionalism in medical practice in Israel. Although conference abstracts and presentations are available online [link to meeting program and abstracts http://www.israelhpr.org.il/e/99/86.htm], it is unfortunate that the sessions were not filmed and that there was no media representation. Shorter workshops focusing on particular issues such as medical education, training of residents, use of digital technologies and their effect on professionalism may be an effective means of involving more Israeli physicians and other health professionals in discussion related to enhancing professionalism in Israel. Future meetings should include patient representatives as well as enhanced participation from younger colleagues, residents and medical students, and inter-professional dialogue. More time should be devoted to discussing work-life balance and enhancing physician satisfaction with their professional lives, as these are clearly related to patient satisfaction and quality of care to [20].

In conclusion, for the first time professionalism in medicine was discussed on a national level in Israel, raising questions but also identifying significant ongoing endeavors to address the subject, especially in medical education. This initial effort engendered a sense commitment from both participants and organizers and should lead to further fruitful undertakings to enhance the experience of both patients and physicians in Israel.

## Additional file

Additional file 1: Workshop Program. (PDF 327 kb)

## Abbreviations

ABIM: American Board of Internal Medicine
ABMS: American Board of Medical Specialties
BGU: Ben Gurion University
BMA: British Medical Association
H-HU: Hadassah-Hebrew University
IMA: Israel Medical Association
MOH: Ministry of Health
MPTS: Medical Practitioners Tribunal Service
NIHPR: National Institute for Health Policy Research
TAU: Tel Aviv University
