# Supplementary material for: International workshop on “professionalism in the practice of medicine- where are we now?”
Source: Isr J Health Policy Res. 2017 Apr 3;6:19. doi: 10.1186/s13584-017-0144-5 (PMC5379495; doi:10.1186/s13584-017-0144-5)
Supplement: Additional file 1: — Workshop Program. (PDF 327 kb) [file 13584_2017_144_MOESM1_ESM.pdf]

THE ISRAEL NATIONAL INSTITUTE FOR HEALTH POLICY RESEARCH

International Workshop
Professionalism in the Practice of Medicine – Where are we now?
December 11-12, 2016

Sunday, December 11th, 2016

09:00-10:00 *Gathering & Welcome Coffee*

10:00-13:00 **OPENING SESSION AND GREETINGS**

Chair: Orly Manor, *Chair, Board of Directors, NIHP*

Moshe Bar Siman Tov, *Director General, Ministry of Health*

Workshop Chair and Introductory Lecture: Ora Paltiel - *Professionalism: What are the issues and why do they need to be discussed? Hadassah-Hebrew University*

Defining Professionalism: International Views

Pali Hungin - *Professionalism: Coping with the changing role of the doctor*, *President, BMA, UK*

Jo Shapiro - *Professionalism: Culture of trust as a key to safety and quality*, *Brigham and Women's Hospital, Harvard University, USA*

Shimon Glick - *Professionalism: Changing views in time and space*, *Ben-Gurion University*

Discussion

13:00-14:00 *Lunch*

14:00-16:10 **Professionalism as a Learned Competency**

Chair: Shmuel Reis, *Hebrew University-Hadassah*

Shiphra Ginsburg - *Fostering professionalism in the education environment*, *Mount Sinai Hospital, University of Toronto, Canada*

Ruti Stashefsky Margalit - *Simulation as a tool to enhance medical professionalism* *Messer-Chaim Sheba Medical Center*

Panel: *Representatives of Israeli faculties of medicine - How do we/should we train physicians in Israel in professionalism?*

Chair: Shiphra Ginsburg, *Mount Sinai Hospital, University of Toronto, Canada*
Peter Gilbey- *Galilee, Bar-Ilan University,* **Shimon Glick-** *Ben-Gurion University,* **Nathaniel Laor-** *Tel-Aviv University,* **Lior Lowenstein-** *Rambam Health Care Campus,* **Shmuel Reis-** *Hebrew University-Hadassah*

Discussion

16:10-16:30 *Coffee Break*

16:30-18:00 **Complaints, Complaints, Complaints. Do they Enhance Professionalism?**

Chair: Avi Israeli, *Ministry of Health*

Shimon Glick, Chaim Hershko, Boaz Lev - *Former and current ombudsmen,* *Ministry of Health*

Panel: *Physicians representing HMO's*
Yair Birnbaum- *Clalit,* **Avi Porath-** *Maccabi,* **Gershon S Segal-** *Leumit,* **Yoav Yehezkelli-** *Meuhedet*

19:45 *Dinner & Evening Program*
Nissan Perez - *Early Photography & Medicine, A brief history,* *Photography historian and curator*

Monday, December 12th, 2016

09:00-10:30 Professional Conduct: Regulation or Self-Regulation. The Carrot and Stick

Chair: Drorith Hochner-Celnikier, ***Hadassah University Hospital***

David Katz - *Professionalism and professional self-regulation: The UK experience*, ***University College London, UK***

Tami Karni - *The IMA: How it handles "lapses" in professionalism,* ***Ethics bureau, IMA***

Discussant: Jo Shapiro - Alternative ways of handling unprofessional behavior

Discussion

10:30-11:00 Coffee Break

11:00-13:00 Professionalism in the Digital Age

Chair: Haim Bitterman, ***Assuta Ashdod Public Hospital***

Shmuel Reis - *Professionalism in the digital age: Required additional competencies, and how they should be acquired,* ***Hebrew University-Hadassah***

Orit Karnieli-Miller - *Our undivided attention? Challenges to professionalism in day-to-day medical encounters,* ***Tel-Aviv University***

Discussant: Pali Hungin

Panel: ***Generation X, Y and Z- How we differ from our teachers and our students***
Chair: Ziv Gil, ***Technion, Israel Institute of Technology***
Sameer Kassem- ***Carmel Hospital,***
Orna Reichman- ***Shaare Zedek Medical Center,***
Rotem Tellem- ***Tel-Aviv Sourasky Medical Center***

Discussion

13:00-14:00 Lunch

14:00-16:30 CLOSING SESSION: THEORY TO PRACTICE

Chair: Ora Paltiel, ***Hadassah-Hebrew University***
Vignettes
Roundtable Chairs:
Amnon Lahad- ***Hebrew University,*** **Dafna Meitar-** ***Tel-Aviv University,***
Dorith Shaham, ***Hadassah-Hebrew University***

Reporters:
A Mark Clarfield- ***Ben-Gurion University,*** **Mordechai Muszkat-** ***Hadassah Mt Scopus,***
Reena Rosenberg- ***Clalit***

Wrap up: Shiphra Ginsburg

Coffee to Go
